# Anaplastic Carcinoma of the Pancreas Mimicking Submucosal Gastric Tumor: A Case Report of a Rare Tumor

**DOI:** 10.1155/2013/523237

**Published:** 2013-12-08

**Authors:** Michimasa Fujiogi, Takashi Kobayashi, Masamichi Yasuno, Michio Tanaka

**Affiliations:** ^1^Department of Surgery, Tokyo Metropolitan Hiroo Hospital, 2-34-10 Ebisu, Shibuya-ku, Tokyo 150-0013, Japan; ^2^Department of Pathology, Tokyo Metropolitan Hiroo Hospital, 2-34-10 Ebisu, Shibuya-ku, Tokyo 150-0013, Japan

## Abstract

Anaplastic carcinoma of the pancreas (ACP) is a rare neoplasm of the pancreas. ACPs are aggressive neoplasms with a poorer prognosis than poorly differentiated ductal adenocarcinomas of the pancreas. The 3-year survival rate of patients with ACP is less than 3%, with a life expectancy of 10 to 20 months. We describe here a 64-year-old man with ACP mimicking a submucosal gastric tumor. The patient was found to have a giant mass mimicking a submucosal tumor. Total gastrectomy with splenectomy and partial resection of the tail of the pancreas were performed. The pathological diagnosis was ACP, with immunohistological findings showing pleomorphic-type ACP. Because the surgery was noncurative, the patient received adjuvant chemotherapy with paclitaxel but died of peritoneal dissemination and multiple liver metastases 4 months after surgery.

## 1. Introduction

Anaplastic carcinoma of the pancreas (ACP) is a rare, aggressive type of pancreatic cancer with a short median survival. Because of its rarity, the clinical presentation, surgical results, and management are not well known, although some Japanese patients have reported long-term survival after surgery. In other patients, however, the tumor is not resectable and chemotherapy is required. Adjuvant chemotherapy has yielded poor survival outcomes. We describe here a patient with ACP who underwent surgery followed by adjuvant chemotherapy with paclitaxel.

## 2. Case Report

**Figure 1:**
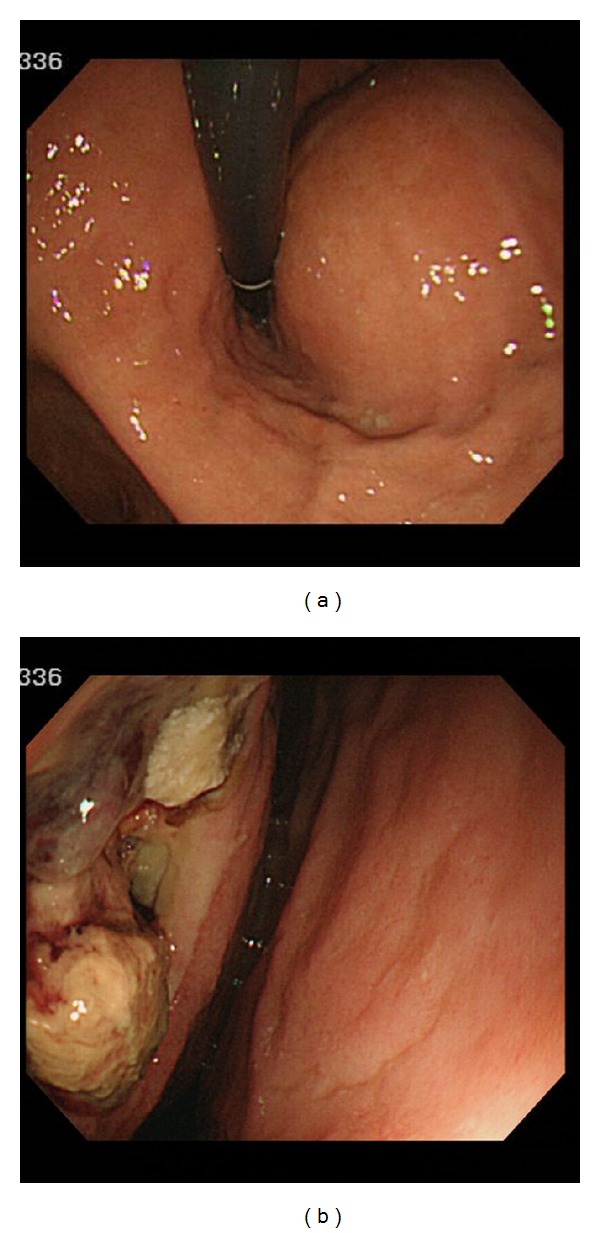
Upper GI endoscopy, showing a giant submucosal-like tumor in the stomach with oozing from an ulceration.

A 64-year-old man was admitted to our hospital with general fatigue and diarrhea. On physical examination, he appeared to be cachexic and a firm mass was palpable on the upper abdomen. Laboratory investigation revealed a slight decline in hemoglobin concentration to 8.2 g/dL and elevated serum carcinoembryonic antigen concentration (67.7 ng/mL). General condition of the patient was poor. Only the upper gastrointestinal endoscopy and plain CT were performed. Upper GI endoscopy revealed a giant lesion within the stomach, located at the posterior wall of the body, showing a submucosal tumor-like appearance with oozing from ulceration (Figure 1). Fiberscopic findings suggested that proper pathologic diagnosis of the submucosal tumor required balling biopsy. However, this was contraindicated in our patient, because of massive bleeding from the tumor, resulting in severe anemia. Upper GI endoscopy revealed that a large mass compressed the gastric wall within the stomach, located at the posterior wall of the body with oozing from ulceration.

**Figure 2:**
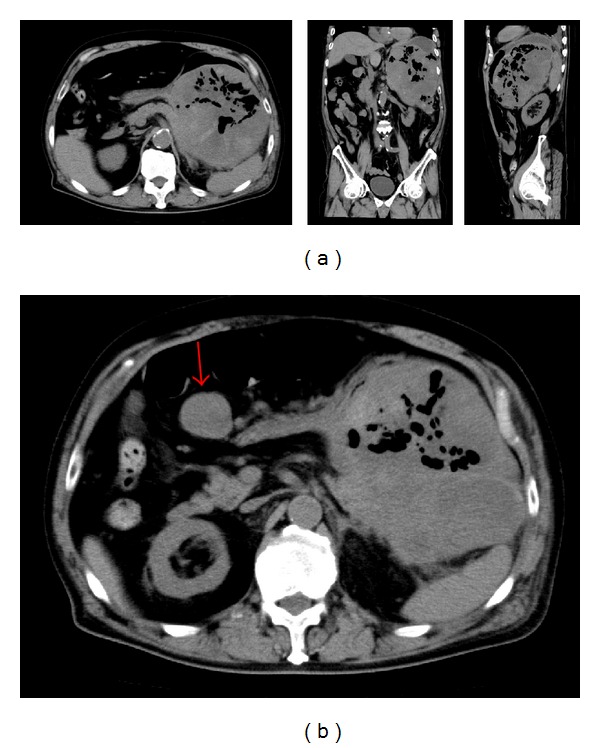
(a) Abdominal computed tomography (CT) scan showing a giant mass with central necrosis in the stomach. (b) Abdominal computed tomography scan showed a peritoneally disseminated lesion, measuring 30 × 30 mm in size (arrow).

A plain CT scan showed a mass with central necrosis in the stomach, with suspected infiltration of the spleen and tail of the pancreas (Figure 2(a)), as well as peritoneal dissemination (Figure 2(b)). In addition, the surface of the mass was smooth, despite the tumor being over 15 cm in diameter. The tumor was diagnosed as a submucosal gastric tumor, which may include gastrointestinal stromal tumor, malignant lymphoma, liposarcoma, and angiosarcoma.

**Figure 3:**
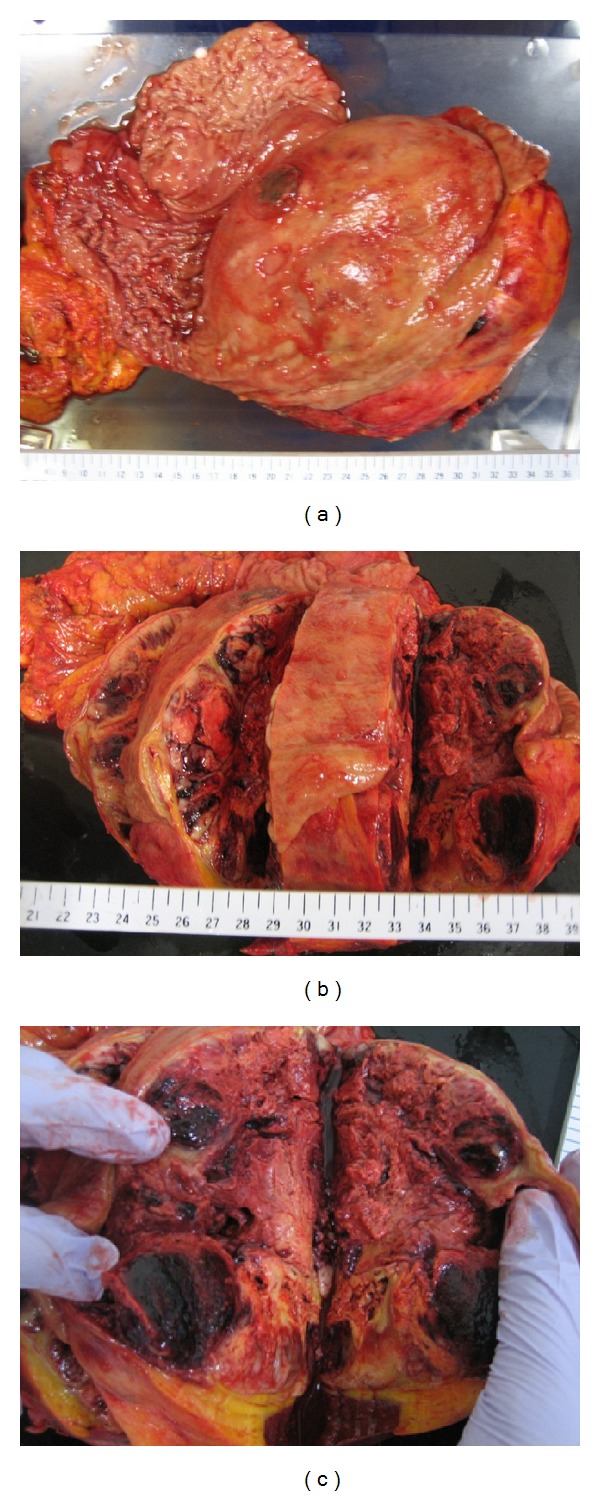
Resected specimen of the giant submucosal tumor-like mass in the stomach. The pathological specimen included the stomach, spleen, part of the pancreas tail, and disseminated lesions. The cut surface of specimen showed necrosis and hemorrhage. On gross examination, the body of the stomach revealed a protruding tumor covered with mucosa and measuring 20 × 18 × 11 cm in size.

**Figure 4:**
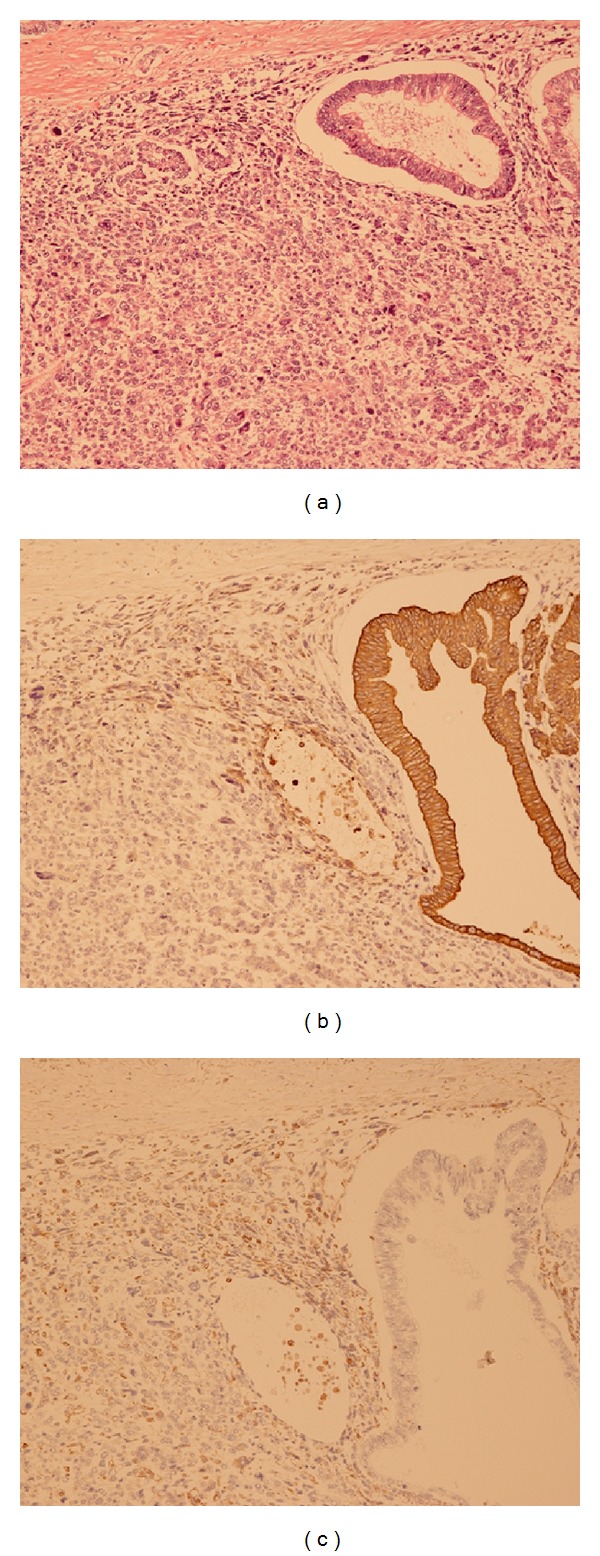
(a) Histologic assessment of the tumor showing the presence of an epithelial component and a nonepithelial sarcomatoid component. The epithelial component showed glandular differentiation, while the sarcomatoid component revealed a complicated growth of spindle-like and oval cells with a pleomorphic and bizarre configuration of the nuclei showing mitosis (HE). (b) Immunohistochemical assays showing that the sarcomatoid component was moderately reactive to antibody against pancytokeratin. (c) The sarcomatoid component was strongly reactive to antibody against vimentin.

The patient underwent a total gastrectomy with splenectomy and partial resection of pancreas tail, accompanied by resection of disseminated lesions. Pathological specimens included the stomach, the spleen, part of the pancreas tail, and the disseminated lesions. Gross examination showed an exophytically-growing tumor in the stomach, measuring 20 × 18 cm (Figure 3) and weighing approximately 3000 g. Microscopically, the tumor consisted of an epithelial component and a non-epithelial, sarcomatoid component. The epithelial component showed glandular differentiation with mucin production, while the non-epithelial, sarcomatoid component showed a complicated growth of spindle-like and oval cells with pleomorphic and bizarre configuration of the nuclei showing mitosis. Both components infiltrated the peripancreatic fat and the sub-mucosal to sub-serosal areas of the stomach wall (Figure 4(a)).

The lesion was assayed immunohistochemically using monoclonal antibodies against pancytokeratin, CEA, CA19-9, vimentin, chromogranin, synaptophysin desmin, *α*SMA, CD34, C-kit, and S-100. The epithelial component was strongly positive for pancytokeratin, CEA and CA19-9; moderately positive for desmin, and negative for vimentin (Figure 4(b)). Positivity for chromogranin and synaptophysin was isolated and scattered. This result was not interpreted as indicative of partial neuroendocrine differentiation but rather as nonspecific.

In contrast, the sarcomatoid component was strongly positive for CEA and vimentin, moderately positive for CA19-9, desmin, and pancytokeratin, weakly positive for cytokeratin, and negative for *α*SMA, CD34, C-kit, and S-100 (Figure 4(c)).

Based on these immunohistological findings, the patient was diagnosed with a pleomorphic-type ACP. Because his surgery was noncurative, he started adjuvant chemotherapy with paclitaxel. However, the patient died of peritoneal dissemination and multiple liver metastases 4 months after the operation.

## 3. Discussion

ACPs, also called “undifferentiated carcinomas” with or without osteoclast-like giant cells, “giant-cell tumors,” and “pleomorphic carcinomas” of the pancreas, are rare malignancies of the pancreas, constituting 0.5–7% of nonendocrine malignancies of the pancreas [1–3].

Clinical symptoms associated with ACP include abdominal pain, body weight loss, anorexia, and back pain, resembling the symptoms of adenocarcinoma of the pancreas. Because the diameter of ACP tends to be greater than that of pancreatic adenocarcinoma, ACPs may be palpated as huge abdominal masses at diagnosis [4, 5]. CT findings include an expansive well-defined margin and a heterogeneous and moderately enhanced mass with central necrosis [6]. In our patient, CT showed a mass with well-defined margins and central necrosis.

The pathogenesis of ACP is unclear, although a recent study of the initial steps in the evolution of early undifferentiated pancreatic carcinoma provided evidence for its ductal origin, suggesting an epithelial origin of the mononuclear undifferentiated cells [7]. These findings suggested that ACP should be considered an anaplastic variant of pancreatic ductal adenocarcinoma [7].

ACPs are aggressive neoplasms with a poorer prognosis than poorly differentiated ductal adenocarcinomas of the pancreas. The 3-year survival rate of patients with ACP is less than 3%, with a life expectancy of 10 to 20 months [2, 4, 8, 9]. Another study reported that median survival was 7.1 months in patients who underwent curative resection compared with 2.2 months in patients who underwent palliative surgery [10]. Because no consensus has been reached on optimal chemotherapy regimens for ACP, little is known about its clinical management. A recent case study reported a complete response to paclitaxel chemotherapy in a patient with unresectable ACP, with paclitaxel selected by chemosensitivity testing [11]. Paclitaxel is a microtubule-stabilizing agent shown in preclinical and clinical studies to be highly effective against several tumor types, including ovarian, breast, and lung cancers. In addition, paclitaxel was recently shown effective against some types of sarcomas, such as carcinosarcoma of the uterus, angiosarcoma, and Kaposi sarcoma, with response rates of 62 to 80%, 56 to 100%, and 62%, respectively, to paclitaxel monotherapy or paclitaxel-containing regimens [12–17]. Despite chemotherapy with paclitaxel, the patient died 4 months after surgery of progressive disease, primarily peritoneal dissemination and hepatic metastasis.

## Abbreviations

ACP: Anaplastic carcinoma of the pancreas
PTX: Paclitaxel
CT: Computed tomography
Upper GI: Upper gastrointestinal endoscopy.

## References

[1] Rosai J, Rosai J (1996). Pancreas and periampullary region. *Ackerman’s Surgical Pathology*.

[2] Paal E, Thompson LDR, Frommelt RA, Przygodzki RM, Heffess CS (2001). A clinicopathologic and immunohistochemical study of 35 anaplastic carcinomas of the pancreas with a review of the literature. *Annals of Diagnostic Pathology*.

[3] Matsuno S, Egawa S, Fukuyama S (2004). Pancreatic cancer registry in Japan: 20 years of experience. *Pancreas*.

[4] Yamaguchi K, Nakamura K, Shimizu S (1998). Pleomorphic carcinoma of the pancreas: reappraisal of surgical resection. *The American Journal of Gastroenterology*.

[5] Chadha MK, LeVea C, Javle M, Kuvshinoff B, Vijaykumar R, Iyer R (2004). Anaplastic pancreatic carcinoma. A case report and review of literature. *Journal of the Pancreas*.

[6] Zamboni G, Capelli P, Pesci A, Beghelli S, Lüttges J, Klöppel G (2000). Pancreatic head mass: what can be done? Classification: the pathological point of view. *Journal of the Pancreas*.

[7] Bergmann F, Esposito I, Michalski CW, Herpel E, Friess H, Schirmacher P (2007). Early undifferentiated pancreatic carcinoma with osteoclastlike giant cells: direct evidence for ductal evolution. *The American Journal of Surgical Pathology*.

[8] Connolly MM, Dawson PJ, Michelassi F, Moossa AR, Lowenstein F (1987). Survival in 1001 patients with carcinoma of the pancreas. *Annals of Surgery*.

[9] Pan Z-G, Wang B (2007). Anaplastic carcinoma of the pancreas associated with a mucinous cystic adenocarcinoma. A case report and review of the literature. *Journal of the Pancreas*.

[10] Strobel O, Hartwig W, Bergmann F (2011). Anaplastic pancreatic cancer: presentation, surgical management, and outcome. *Surgery*.

[11] Wakatsuki T, Irisawa A, Imamura H (2010). Complete response of anaplastic pancreatic carcinoma to paclitaxel treatment selected by chemosensitivity testing. *International Journal of Clinical Oncology*.

[12] Skubitz KM, Haddad PA (2005). Paclitaxel and pegylated-liposomal doxorubicin are both active in angiosarcoma. *Cancer*.

[13] Schlemmer M, Reichardt P, Verweij J (2008). Paclitaxel in patients with advanced angiosarcomas of soft tissue: a retrospective study of the EORTC soft tissue and bone sarcoma group. *European Journal of Cancer*.

[14] Tulpule A, Groopman J, Saville MW (2002). Multicenter trial of low-dose paclitaxel in patients with advanced AIDS-related Kaposi sarcoma. *Cancer*.

[15] Fardet L, Stoebner P-E, Bachelez H (2006). Treatment with taxanes of refractory or life-threatening Kaposi sarcoma not associated with human immunodeficiency virus infection. *Cancer*.

[16] Toyoshima M, Akahira J-I, Matsunaga G (2004). Clinical experience with combination paclitaxel and carboplatin therapy for advanced or recurrent carcinosarcoma of the uterus. *Gynecologic Oncology*.

[17] Pectasides D, Pectasides E, Papaxoinis G (2008). Combination chemotherapy with carboplatin, paclitaxel and pegylated liposomal doxorubicin for advanced or recurrent carcinosarcoma of the uterus: clinical experience of a single institution. *Gynecologic Oncology*.

